# The biotechnological potential of proteases from hematophagous arthropod vectors

**DOI:** 10.3389/fcimb.2023.1287492

**Published:** 2023-10-26

**Authors:** Carla Nunes de Araújo, Paula Beatriz Santiago, Giulia Causin Vieira, Gabriel dos Santos Silva, Renan Pereira Moura, Izabela Marques Dourado Bastos, Jaime Martins de Santana

**Affiliations:** ^1^ Host-Pathogen Interface Laboratory, Department of Cell Biology, Institute of Biology, University of Brasília, Brasília, DF, Brazil; ^2^ Faculty of Ceilândia, University of Brasília, Brasília, DF, Brazil

**Keywords:** protease, arthropod vector, hematophagous vector, biothechnological potential, protease-based therapy

## 1 Introduction

Vector-borne diseases contribute to over 17% of total infectious diseases (WHO, 2023). Mosquitoes, ticks, fleas, sand flies and triatomines are hematophagous arthropod vectors of debilitating pathogenic microorganisms that cause widespread human infectious diseases, such as malaria, zika, dengue fever, yellow fever, Japanese encephalitis, tick-borne encephalitis, Lyme disease, plague, rickettsiosis, leishmaniasis and Chagas disease (WHO, 2023). Scientists worldwide have been making significant contributions to the field of parasitic infections transmitted by arthropod vectors investigating their molecular biology. Arthropod proteases play essential roles in their blood-feeding behavior, egg development, and immunity (Santiago et al., 2017). Proteases are multifunctional enzymes that hydrolyze one or more peptide bonds in a protein or peptide. Their activity can result in modification/activation/inactivation of proteins, enzymes and peptides, protein targeting, and amino acids recycling (Rawlings and Salvesen, 2013). Due to their inherent involvement in many key physiological processes, inhibition or abnormal enzyme production or secretion can lead to various pathological conditions (López-Otín and Bond, 2008).

Otherwise, the remarkable substrate affinity and specificity of proteases are features that make enzyme therapy an important approach for treating/managing multiple ailments (Shankar et al., 2021). In fact, the accumulated knowledge on the catalytic and functional diversity of proteases has driven the development of therapeutic approaches for cardiovascular disease, inflammation, sepsis, digestive and retinal disorders, among others (Craik et al., 2011), and also their incorporation into dermatological products (Del Rosso, 2013). Several protease therapies have been approved by the U.S. Food and Drug Administration, and many are in clinical development (Badalamente and Hurst, 2007; Thomas and Bayat, 2010; Ranieri et al., 2012; Gelbard et al., 2013; Brunengraber et al., 2014; Lyden et al., 2019; Jadhav et al., 2020; Kaufman-Janette et al., 2021; Tamimi et al., 2021; Obed et al., 2022).

Our previous review covered contemporary advances in the proteases from hematophagous arthropod vectors up to 2016 (Santiago et al., 2017). In this opinion article, we summarize further research findings on vector proteases and emphasize their biotechnological potential for the development of innovative protease-based drugs with broad clinical applications. This potential arises from the considerable effort that has been made by high-throughput transcriptomic and proteomic approaches to catalog arthropod vector proteins, followed by structural biology and protease activity investigations.

## 2 Finding the molecule and its biological activity

The progress in biotechnology and the application of high-throughput sequencing technologies have unveiled a remarkable number of proteases in hematophagous arthropod vector tissues, giving rise to an emerging field of scientific exploration. Hematophagous consume blood from vertebrate hosts as a nutrient source. Their ability to locate the prey, their behavior, their mouthparts morphology, and their physiology are an interesting combination of tools very well adapted to obtain blood meals. Once the host is found at the right place and time, the hematophagous pursue their meal boldly. During the bite, the host suffers tissue and vascular injuries, which trigger a series of interrelated mechanisms, such as hemostasis, inflammation, and immune responses (Ribeiro, 1987; Ribeiro, 1995). Large-scale sialotranscriptomic (salivary glands transcriptome) and sialoproteomic (salivary glands proteome) analyses have been reported for various blood-feeding arthropods (Andersen et al., 2007; Arcà et al., 2007; Calvo et al., 2007; Assumpcao et al., 2008; Chmelar et al., 2008; Andersen et al., 2009; Alves-Silva et al., 2010; Schwarz et al., 2014; Santiago et al., 2016; Santiago et al., 2018; Praça et al., 2021) disclosing the saliva of hematophagous is indeed a potent pharmacologically active fluid capable of counteracting the hemostatic, inflammatory, and immune responses of the vertebrate host (Ribeiro, 1987; Ribeiro, 1995). The comprehensive mapping of already reported sialomes (salivary glands transcriptomic and proteomic analyses) revealed different protease families are produced by salivary glands cells (Andersen et al., 2007; Arcà et al., 2007; Calvo et al., 2007; Assumpcao et al., 2008; Chmelar et al., 2008; Andersen et al., 2009; Alves-Silva et al., 2010; Schwarz et al., 2014; Santiago et al., 2016; Santiago et al., 2018; Praça et al., 2021).

Metalloprotease and serine protease sequences have been disclosed in the salivary glands of ticks (Valenzuela et al., 2002; Harnnoi et al., 2007; Decrem et al., 2008a) and triatomines (Santiago et al., 2016; Santiago et al., 2018), and their functions are under investigation. Metalloprotease members are known to inhibit platelet aggregation and hydrolyze fibrinogen and fibronectin preventing blood clotting (Huang et al., 1993; Feitosa et al., 1998; da Silveira et al., 2007; Hsu et al., 2007; Hsu et al., 2008; Trevisan-Silva et al., 2010). Interestingly, metalloproteases are abundant in snake venoms showing important antithrombotic and hemorrhagic activities (Gutiérrez et al., 2005; Sajevic et al., 2011). In agreement, functional studies of Metis 1 and Metis 2, two metalloproteases found in the salivary glands of *Ixodes ricinus* ticks, have shown that these proteins play a significant role in regulating fibrinolysis (Decrem et al., 2008b). Although the exact role of these components in saliva remains unknown, it is important to explore their potential in producing peptides that can specifically target inflammation and coagulation cascades (Amino et al., 2001). This could lead to the development of more effective and precise protease-based drugs, which could be used to treat hemorrhagic and thrombotic disorders, as well as cardiovascular and cerebrovascular diseases by preventing thrombus formation.

During the digestion process of blood components in the gut of hematophagous organisms, proteases function within a network of multiple enzymes that break down hemoglobin. These multi-peptidase repertoires are mainly composed of serine proteases in mosquitoes and cysteine and aspartic proteases in ticks and triatomines (Santiago et al., 2017). *Trypanosoma cruzi*, the causative agent of Chagas disease, proliferates and develops inside the intestines of triatomine vectors. As part of the feeding process, while consuming blood, the vector releases *T. cruzi* contaminated feces onto the skin of the vertebrate host. The protozoan can infect the host through the bite injury or intact mucosae. In this context, it has been suggested that *T. cruzi* can modulate insect metabolism, increasing the activity levels of digestive enzymes (Borges et al., 2006; Buarque et al., 2013). The investigation of protease activity in *Rhodnius prolixus* triatomine unveiled distinct sequential patterns of protease expression within the insect digestive system, including cathepsin L-like and cathepsin D-like proteases (**Table 1**) (Henriques et al., 2020). It is important to conduct comprehensive studies to better understand the role of proteases in the trypanosome/vector interaction. We must consider whether *T. cruzi* modulation of digestive enzymes could enhance the infection process in humans. From this knowledge, strategies for direct intervention in the vector gut physiology or the modulation of interactions between the pathogen and digestive enzymes may be developed. This hypothesis is an attractive area of research, as the digestive enzymes produced by triatomines could potentially be used as a parasite control intervention strategy.

**Table 1:** Novel information on members from the serine, cysteine and aspartic protease families disclosed in triatomine, tick and mosquito tissues from 2017 to 2023.

Family	Protease	Vector	Localization	Role	Ref
**Serine**	Trypsin-like (Triapsin)	*Triatoma infestans*	Salivary glands	Vasodilation	(Oliveira et al., 2021)
	Trypsin-like (Is- coding sequences)	*Ixodes scapularis*	Gut	Digestion	(Reyes et al., 2020)
	Trypsin (T714)	*Aedes aegypti*	Gut	Immunity	(Angleró-Rodríguez et al., 2017)
	Chymotrypsins (Ag-coding sequences)	*Anopheles gambiae*	Female reproductive tracts	Mating	(Bascuñán et al., 2020)
	Chymotrypsins (MatRAP1)	*Anopheles gambiae*	Female reproductive tracts	Mating	(Bascuñán et al., 2020)
	Chymotrypsins (AaCT-1)	*Aedes aegypti*	Hemolymph	Immunity	(Zhu et al., 2021)
**Cysteine**	Cathepsin L (Rp-activity)	*Rhodnius prolixus*	Gut	Digestion	(Henriques et al., 2020)
	cathepsin L-like (AaCatL)	*Aedes aegypti*	Salivary glands, Gut	Immunity	(Oliveira et al., 2020)
	Cathepsin B (Hl- coding sequences)	*Haemaphysalis longicornis*	Eggs	Vitellin degradation	(Zhang et al., 2019)
**Aspartic**	Cathepsin D (Rp-activity)	*Rhodnius prolixus*	Gut	Digestion	(Henriques et al., 2020)
	Cathepsin D (Hl- coding sequences)	*Haemaphysalis longicornis*	Eggs	Vitellin degradation	(Zhang et al., 2019)

Yet concerning triatomine proteases, triapsin (**Table 1**), a serine protease from the saliva of *Triatoma infestans* first described in 2001 (Amino et al., 2001) and still under investigation, is capable of inducing hydrolysis of protease-activated receptors (PARs), with a distinct preference for cleaving the PAR-2 peptide. Mass spectrometry analysis has corroborated the presence of a single cleavage site, corresponding to the activation site of the PAR-2 receptor. Moreover, nitric oxid (NO) levels measurements have demonstrated that triapsin induces a dose-dependent release of NO in cultured human umbilical vein endothelial cells. NO appears to play a role in the vasorelaxant activity of triapsin. Furthermore, observations of increased mouse ear venular diameter following triapsin exposure suggest a plausible link between triapsin activity mediated by PAR-2 and vasodilation induced by *T. infestans s*aliva (**Table 1**) (Oliveira et al., 2021).

While consuming blood, ticks are capable of transmitting viral and bacterial diseases, such as tick-borne encephalitis caused by the tick-borne encephalitis virus, and Lyme disease caused by *Borrelia burgdorferi* spirochetes (Nuttall, 1999). Ticks have a digestive system that consists of a combination of cysteine-aspartic proteases, which operate together in hemoglobinolysis (Sojka et al., 2008). Previous studies have highlighted the significance of IrCD 1-3, which are three different isoforms of cathepsin, in playing various biological roles in *Ixodes ricinus* tick. These enzymes are expressed not only in gut cells but also in salivary glands and ovaries, and may generate antimicrobial peptides, that aid in immune responses against foreign invaders (Sojka et al., 2012; Sojka et al., 2016). Of interest, in ticks, trypsin-like serine proteases may participate in the liberation of dipeptides and free amino acids in the intracellular midgut vesicles and outside the digestive vesicles (Horn et al., 2009). In *Ixodes scapularis*, vector of Lyme disease, trypsin levels increase significantly after repletion. Knockdown of tick serine proteases was shown to lower hemoglobin degradation and negatively impacted levels of active trypsin in the midgut of *I. scapularis*, as well as blood feeding, survival, and fecundity in this species (**Table 1**) (Reyes et al., 2020). Another potential candidate for investigation is the vitellin degrading cysteine endopeptidase (VTDCE), found in *Boophilus microplus* tick eggs. This enzyme plays a role in both vitellogenesis and embryonic development (Seixas et al., 2003). VTDCE also possesses antimicrobial activity, particularly reported against *Staphylococcus epidermidis* (Oldiges et al., 2012). Proteases like IrCD and VTDCE hold significant biotechnological potential as therapeutic agents for pathogen control. More recently, it was also shown that vitellin degradation in *Haemaphysalis longicornis* eggs involves three enzymes: cathepsin B, cathepsin D, and acid phosphatase (**Table 1**) (Zhang et al., 2019).

In mosquitoes, it was demonstrated that the reproductive success of *Anopheles gambiae*, an important vector of the malaria parasite *Plasmodium* spp, relies on a single copulation event after which most females become permanently refractory to further mating. In females, two chymotrypsin‐like serine proteases regulated by the male‐synthetized steroid hormone 20‐hydroxyecdysone (20E) play an important role in modulating their susceptibility to mating. The depletion of the Mating Regulated Atrial Protease 1 (MatRAP1), one of these proteases, by RNA interference, reduced female refractoriness to further copulation, allowing a significant proportion of females mate again (**Table 1**) (Bascuñán et al., 2020). In *Aedes aegypti*, a cathepsin L-like peptidase (AaCatL) was cloned, expressed, purified, and biochemically characterized. Transcripts of AaCatL were detected in the salivary glands and midgut from *Ae. aegypti* and seem to be negatively correlated with DENV-2 virus titers, indicating AaCatL may have a role during mosquito-DENV interactions. Purified recombinant AaCatL has a typical cathepsin L-like substrate profile. Authors suggest AaCatL may inhibit the activation of caspases (**Table 1**) (Oliveira et al., 2020). In field-caught *Ae. aegypti*, the gut-associated fungus *Talaromyces* was shown to profoundly down-regulate digestive enzyme genes and trypsin activity in the mosquito and to render *Ae. aegypti* more permissive to DENV infection (**Table 1**) (Angleró-Rodríguez et al., 2017). Interestingly, it was reported that the expression of some chymotrypsins from *Ae. aegypti* and *Aedes albopictus*, such as AaCT-1 (**Table 1**) are suppressed by the human blood-derived microRNA hsa-miR- 150-5p, enhancing DENV and ZIKV loads in these mosquitoes (**Table 1**) (Zhu et al., 2021).

**Table 1** presents novel information on eleven members from the serine, cysteine and aspartic protease families disclosed in triatomine, tick and mosquito tissues, from 2017 to 2023. For a list of proteases from hematophagous arthropod vectors disclosed before this period, we suggest the information that was written in our previous publication (Santiago et al., 2017).

## 3 Maxadilan, the vasodilator from sand flies

Turning to the clinical applications of proteins from hematophagous arthropod vectors, although not a protease, a potent vasodilator peptide, named maxadilan, from the salivary glands of *Lutzomyia longipalpis*, the sand fly that transmits *Leishmania* spp., selectively and potently activate the PAC_1_ receptor (Lerner et al., 2007), following intradermal injection (Marynissen et al., 2022). This receptor is activated in the pathophysiology of migraine. Maxadilan was proposed to be used as a novel pharmacodynamic biomarker for the early clinical development of PAC_1_ receptor antagonists (Marynissen et al., 2022), highlighting the potential clinical use of proteins from hematophagous arthropod vectors.

## 4 The ongoing challenge

Developing new therapies for infectious diseases transmitted by arthropod vectors, as well as for controlling disorders that affect platelet function and blood clotting is a considerable challenge. Understanding the functional role of proteins is a significant task of the post-genome research era. The journey from discovering a promising compound to progressing to human clinical trials and reaching the market spans many years and entails substantial financial investments. However, these paths begin with fundamental science. In this opinion article, we wanted to emphasize the biotechnological potential of hematophagous vector proteases. Realizing this potential requires a deeper research interest in this area and a commitment to developing effective methodologies for structural and functional studies.

## Author contributions

CA: Conceptualization, Project administration, Writing – review & editing. PS: Conceptualization, Project administration, Writing – review & editing. GC: Writing – original draft. GS: Writing – original draft. RM: Writing – original draft. IB: Writing – review & editing. JS: Writing – review & editing.
